# Nursing staff and supervisors perceptions on stress and resilience: a qualitative study

**DOI:** 10.1186/s12912-025-02712-x

**Published:** 2025-01-22

**Authors:** Madeleine Helaß, Anja Greinacher, Melanie Genrich, Andreas Müller, Peter Angerer, Harald Gündel, Florian Junne, Christoph Nikendei, Imad Maatouk

**Affiliations:** 1 Department of General Internal Medicine and Psychosomatics, University Hospital Heidelberg, Im Neuenheimer Feld 410, Heidelberg, 69120 Germany; 2 Clinical Psychology, Interaction and Psychotherapy Research, Faculty of Social Sciences, University of Mannheim, Mannheim, 68159 Germany; 3 Institute of Psychology, Work and Organizational Psychology, University of Duisburg-Essen, Essen, 45141 Germany; 4 Faculty of Medicine, Institute of Occupational, Social and Environmental Medicine, Centre for Health and Society, Heinrich Heine University Düsseldorf, Düsseldorf, Germany; 5 Clinic of Psychosomatic Medicine and Psychotherapy, University Hospital Ulm, Albert-Einstein-Allee 23, Ulm, 89081 Germany; 6 Department of Psychosomatic Medicine and Psychotherapy, University Magdeburg, Leipziger Straße 44, Magdeburg, 39120 Germany; 7 Section of Psychosomatic Medicine, Psychotherapy and Psychooncology, Department of Internal Medicine II, Julius-Maximilian University Würzburg, Oberdürrbacher Str. 6, Würzburg, 97080 Germany

**Keywords:** Nurses, Supervisors, Stress, Resiliencies, JDR, Transactional model, Qualitative approaches

## Abstract

**Background:**

Supervisor–subordinate relationship is high relevant in dealing with work-related stress and providing a compassionate, high-quality, and safe nursing care while meeting the needs of the hospital. Our aim was to assess the predisposing risk and resilience factors of the stress of nursing staff as well as to explore the common and distinctive perceptions of these factors between nurses without a managerial position (nursing staff) and employees in a supervising position (nurse managers, ward nurses).

**Design:**

Generic qualitative study using half-standardized interviews.

**Methods:**

Fifty nurses and supervisors from different departments from a German hospital of maximum medical care participated in this study between August and November 2018. Nineteen face-to-face interviews and five focus groups were conducted. Transcripts were subjected to structured qualitative content analysis.

**Results:**

Systematised in Lazarus’s transactional model, nurses, and supervisors mentioned similar risk and resilience factors of stress. Disagreement in suggested responsibility for nurses’ stress or health and an evaluation of implemented measures meeting the nurses’ needs are discussed.

**Conclusion:**

Nursing staff and supervisors should enforce exchange to reduce disagreements in perceptions and to improve mutual understanding. Furthermore, measures to meet nurses’ needs to minimize stress and to improve collaboration and job satisfaction should be developed in close coordination with the target group. The focus should be placed on restructuring training and education programs with supplementation of self-responsibility promotion.

**Trail registration:**

The study was registered with the German Register for Clinical Studies (DRKS 00013482) on 09 March 2018.

**Supplementary Information:**

The online version contains supplementary material available at 10.1186/s12912-025-02712-x.

## Background

 In the 21st century, the profile of employment of nurses altered due to demographic development as well as fundamental technological, social and structural changes in terms of qualification (e.g., academisation), breaking up of traditional occupational fields (e.g., new medical fields and task areas) and biographies (e.g., several job changes until retirement), whereas conventional securities (e.g., job securities) decreased. These work-related demands put a strain on nurses’ resources, resilience and coping strategies. The growing gap between increasing strains and failure to compensate for them can result in health problems, low workability and increased intent to leave [1, 2]. Moreover, the pandemic situation has exacerbated the fragility of our health care systems and their workforce [3].

Supervisors, as a superior authority to the nursing staff, play a decisive role [4] in creating a healthy work environment, but there is scarce research on nurses’ stress-related phenomena from the perspective of supervisors. The present study aims to fill this gap by evaluating the factors of stress and resilience from the perspective of nurses and supervisors and by clarifying the similarities and differences in the perceptions of both groups.

In a pressurised health system, nursing staff must face constant and consistently high expectations and work demands due to patient-related, professional, environmental, organisational and personal factors [5]. Patient-related factors concern, amongst others, uncertainty regarding treatment, demands of both patients and their families and dealing with death and dying [6]. Insufficient professional relationships and communication with colleagues and supervisors can increase the nurses’ stress [7]. In addition to environmental factors such as staff shortages and insufficient time for the work load, there are also organisational factors, including lack of support, workforce and material [8]. All of these factors may lead to work-related stress, job dissatisfaction [9], low workability [10], and poor mental health [11]. This can manifest in emotional exhaustion and burnout [2, 12], compassion fatigue [13] and, finally, intent to leave [1]. Currently, one of the most disastrous consequences for the health care system is a high turnover rate, as it exacerbates the precarious situation of nursing shortage [14].

The job-demand and resource model [15] is a well-established and widely used theoretical framework for stress in the nursing profession [16, 17]. This model assumes job demands and resources as “physical, psychological, social and organizational aspects” [15]. Job demands require effort with associated costs and job resources are suggested as functional in achieving work goals, reducing job demands and the associated costs [15]. Recent studies found correlation of high job demands with nurses burnout [18] and turnover intentions [19].

A good supervisor–subordinate relationship (SSR) can be a job resource when dealing with work-related stress. The SSR affects nurses’ trust in their supervisors and their perception of supervisor support and can maintain and improve workability [20]. The SSR is positively related to well-being, job satisfaction [4, 21] and to positive outcomes for organisations (leader member exchange theory (LMX-Theory), [22]). Supervisor support as an aspect of SSR plays a crucial role in creating a healthy working environment, although positive SSR consequently promotes organisational commitment behaviour on the part of nurses, resulting in greater organisational effectiveness and a significant reduction in turnover [4] at least. In contrast, a low level of social support from supervisors combined with a high workload is associated with emotional exhaustion and a depressive state of nurses [23] and is negatively related to nurses’ staying in their job [24]. Overall, to provide compassionate, high-quality, and safe nursing care while meeting the needs of the hospital, a healthy environment with an supportive relationship with supervisors seems to be necessary [25].

There is growing body of literature on stress, stress management, resilience in the nursing profession and supervisor relationship or support. However, to our knowledge, there is no research addressing registered nurses’ work-related stress and resilience factors from the perspective of supervisors as compared with the perspective of nursing staff.

## Methods

### Aims

This study aimed to assess the predisposing risk and resilience factors of the stress of nursing staff as well as to explore the common and distinctive perceptions of these factors between nurses without a managerial position (nursing staff) and employees in a supervising position (nurse managers, ward nurses).

### Design

The study reports parts of the needs assessment within the subproject “Healthy Ageing in the Nursing Profession” (“Gesund Altern im Pflegeberuf”). This subproject was one of five subprojects of the project “Mental Health in the Hospital Workplace” (“Seelische Gesundheit am Arbeitsplatz Krankenhaus, SEEGEN”) [26] funded by the General Ministry of Research and Education.

In the needs assessment of “Healthy Ageing in Nursing Profession”, participants were asked about several topics, age stereotypes (already published in Helaß et al. [27]) and stress/resilience factors, which were surveyed independently of each other and evaluated separately, so that no overlaps or thematic mixing could occur. This study followed a generic qualitative approach [28], as the participants were not only asked about their own views, but also the supervisors views of (other) nursing staff. Interviews and focus groups were conducted at a university hospital of maximum medical care (surgical, conservative and mental health departments) in Germany.

### Participants

The participants were registered nurses, ward nurses and nurse managers of a German university hospital.^1^ All registered nurses and supervisors were addressed to take part in the study. For the study, ward nurses and nurse managers were grouped together as “supervisors”. Of 3090 contacted nurses and supervisors, *n* = 50 participants from nine departments took part in the study (response rate = 1.62%), as described in Helaß [27]. Table 1 presents the sociodemographic data of the participants.

**Table 1:** Sociodemographic data^a^

		Interviews				Focus groups				Total
		*RN* *n (%)*	*WN* *n (%)*	*NM* *n (%)*	*Total* *n (%)*	*RN* *n (%)*	*WN* *n (%)*	*NM* *n (%)*	*Total* *n (%)*	
		9 (48)	5 (26)	5 (26)	19 (38)	14 (45)	13 (42)	4 (21)	31 (62)	50 (100)
Gender										
Male		4 (44)	3 (60)	2 (40)		8 (57)	5 (38)	2 (50)		24 (48)
Female		5 (56)	2 (40)	3 (60)		6 (43)	8 (62)	2 (50)		26 (52)
		*RN*	*WN*	*NM*	*Total*	*RN*	*WN*	*NM*	*Total*	
					19 (38)				30 (60)^b^	49 (98)
Age, y										
M		38.00	46.20	52.20	43.89	50.14	49.83	51.33	50.14	47.39
SD		11.83	7.19	6.10	10.95	12.48	8.17	8.14	12.48	10.89
Range		23–57	35–52	42–58	23–58	22–63	34–59	42–57	22–63	22–63
Median		38	39	54	46	55	48	55	55	51
Professional experience, y										
M		17.33	15.60	24.20	18.68	26.90	12.92	20.67	21.00	20.10
SD		12.08	8.17	12.15	11.16	13.07	7.45	9.02	12.86	12.16
Range		4–38	3–25	3–32	3–38	3–40	1–30	12–30	1–40	1–40
Median		20	15	30	20	31	14	20	19	20

^a^*M* Mean, *SD* Standard deviation, *RN* Registered nurse (no managerial position), *WN* Ward nurse (registered nurse with management responsibility for one ward, superior to registered nurses), *NM* Nurse manager (superior to ward nurses and responsible for departments or a group of wards)

^b^Age and professional experience were collected from 49 participants; one focus group participant did not provide any information (*n* = 30)

Following theoretical sampling [29], the selection of the participants was initially open but was adjusted in the process of data evaluation by specifically selecting based on sociodemographic factors [30] to achieve an almost equal distribution in terms of gender and hierarchical level and the most heterogeneous distribution possible in terms of age, professional experience and hospital affiliation [31].

Either interviews or focus groups were conducted with the 50 participating nurses. The focus groups were conducted as a supplement to the 19 face-to-face interviews in order to work out common points of view at hierarchical level and to clarify disagreements. To verify this, an attempt was made to conduct two focus groups per hierarchy level. 31 nursing staff were assigned to the focus groups according to their hierarchical level, so that 5 focus groups were held (two focus groups of registered nurses, two of ward nurses, and one of nurse managers). One participant withdrew his written consent.

### Data collection

A literature-based semistructured interview guide of 16 questions on work situation and professional biography was constructed according to the model of Lazarus` dichotomy of risk factors and resources [32]. It was used for face-to-face-interviews and focus groups (see Appendix A), which were conducted in the German language between August and November 2018 in the offices of the research team or nurse managers. The mean duration of the interviews was 32 min (range, 26–42 min) and of focus groups 33 min (range, 30–38 min). One moderator (MH) led the hierarchical-level–grouped focus groups. The participants were encouraged to comment and directly asked questions towards each other [33]. In addition, the mention of physical and psychological complaints was quantified.

### Ethical consideration

Written informed consent was obtained. The Ethics Committee of Heidelberg University reviewed and approved the study (S-005/2018). The study was registered with the German Register for Clinical Studies (DRKS 00013482) on 09 March 2018.

### Data analysis

The face-to-face interviews and focus groups were transcribed and analysed by two researchers (MH and IM) according to Mayring’s qualitative content analysis [34] using MAXQDA Analytics Pro 2018 [35]. The analysis was conducted inductively in order to find the most important attitudes of the participants and to identify core variables. The preliminary category system that emerged from the analysis of 10 transcripts was further refined and verified by analysing the other interviews. The completed category system was checked again on the first 10 transcripts and the semantic units were re-categorised where necessary. This process was continuously discussed by the research team until consensus and data saturation [36] were reached. The similarities and differences of the reports of supervisors (SU, ward nurses and nursing managers) and registered nurses (RN, nurses without managerial position) are described for each category.

### Validity and reliability/rigour

The current research standards (recommendations of Lincoln and Guba (1986) and the Consolidated Criteria for Reported Qualitative Research (COREQ)) were adhered to and followed in terms of credibility, reliability, confirmability, authenticity and transferability. Extensive notes and self-reflections on experiences and biases toward the research subject discussed in the research team [37] were intended to enhance credibility.

## Results

### Findings of the content analysis

**Fig. 1:**
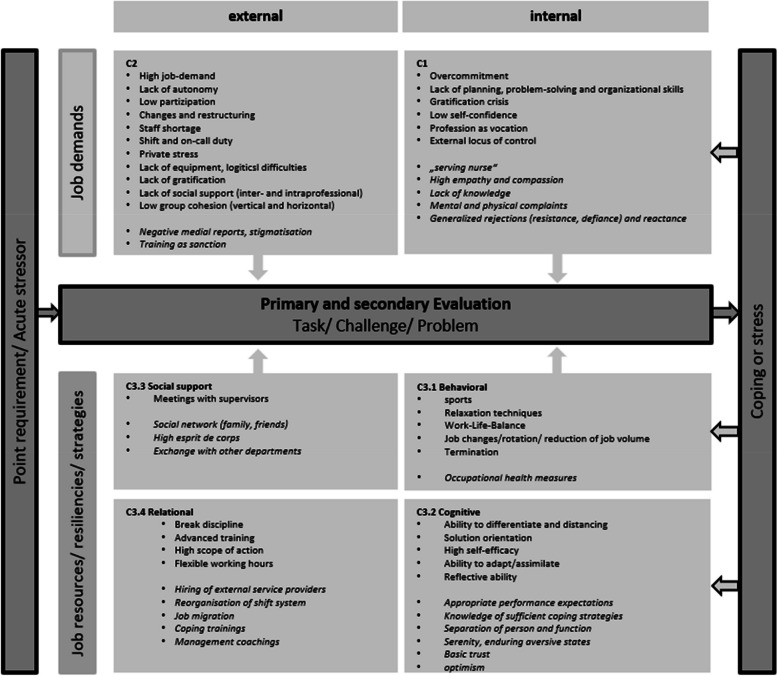
Model of job stress and resilience in nursing. Note: Based on the job-demand and resources-model, integrated in the transactional model of Lazarus, a constellation of conditions for nurses emerged from our results. Congruent opinions of nurses and supervisors were not italicized. Divergent opinions are shown in italics

In the qualitative analysis, 594 single codes were identified, from which three main categories and nine subcategories were formed. Category 1 summarises job demands as intrapersonal risk factors and predispositions of stress and health complaints in the nursing profession (C1, 93 codes). External job demands are described in category 2 (C2, 284 codes) on five subcategories (high job demand, lack of autonomy, economical and relational factors, lack of gratification, and lack of social support). Category 3 includes job resiliencies, strategies and resources on four subcategories (C3, 217 codes): behavioural, cognitive, social, and relational. Table 2 provides the category system, including definitions for each main category with (an) exemplary citation(s). Figure 1 shows the job demands and resources factors embedded in the transactional stress model [32]. This model assumes cognitive perspective on the development of stress reactions—not the objective situation per se (i.e., work demands and expectations), but rather the appraisal of the situation based on available resources and coping strategies seems to be the determining factor for stress development. In the case of a confrontation with an acute stressor or a punctual demand, a primary evaluation of the stressor occurs on the basis of available resources and strategies: existing risk factors are weighed against the available resources. If there is a predominance of resources and resilience, the punctual demand can be regarded as a task. If risk and resilience balance each other out, the requirement is seen as a challenge. However, if risk factors outweigh resources and strategies, a stress reaction occurs and active coping becomes necessary. A successfully mastered challenge has an effect on both internal risk factors and resources, in that the former are reduced due to the positive learning experience and the latter are strengthened. Research on Lazarus’s stress model and the nursing profession supports this thesis: stress depends on employees’ individual coping strategies based on qualification, social competence, and subjectively estimated control possibilities with regard to the critical situation and the experienced support [38].

**Table 2:** Category system

Key Domains	Subdomains	Definition	Quotation
C1 Intrapersonal risk factors/predispositions And Health Complaints		• Factors that are inherent in an individual (behavioural factors) and are related to occupational stress • Complaints and preexisting diseases described in connection with the occupational stress	… which of course also brings with it a certain tension for us or demands even more overview, especially from us in nursing … perhaps also an ability to act with a bit of foresight. (RN_18)
C2 External risk factors/environmental conditions	C2.1 High job demand	• Long working hours, overtime • Shift work, call/standby duty • Little time for recovery or recovery does not last long • Higher retirement age/longer working life • Additional tasks (training of new nurses and doctors) • New areas of responsibility (e.g., taking over medical activities, puncturing a port catheter) • High responsibility for new employees • Area care with a high number of patients and various clinical pictures • Inpatients with high care costs and more intensive therapies than out-patients • Extensive care of the relatives • Enduring the suffering of patients • Patients and relatives with a migration background and high demands on nursing staff • Violence • High documentation effort • Peak hours • Short-term change of activity (helping out on other wards) • Lack of knowledge for new work areas due to few opportunities to attend further training courses • Double burden due to further training parallel to ward duty • Private stress and second job Rule: concrete consequences of economisation/personnel shortage are named; facts or description of the circumstances of economisation/personnel shortage to K2.3 Economical factors	What’s changed now is that the interns are doing a rotation. That everyone has to spend 3 months in the operation room with us. Which of course also means that we have to train them a bit every time, even if it’s not our direct job. So, there are other interns who show up, but they are often not there. And then of course we have the task of always showing, that’s how it is done. (RN_I6)
	C2.2 Lack of autonomy	• Low information flow from management and other call groups • No say • Little leeway • External control/foreign control Rule: focus is the dependence on other occupational groups; circumstance must be mentioned in connection with a direct burden and must not be mediatised about working climate	To work away what has to be worked away, so that you are free for what is coming, so I think the worst shifts are the ones where you don’t act but only react to what is happening. You have your timetable for the day what you are going to do and then there are always events that mess up your timetable and those are the worst shifts, but you can’t influence that. One measure is of course just to watch, to get the To Do’s over as fast as possible in order to react to what’s coming. (RN_FG2)
	C2.3 Economical and relational factors/external impact	Clinics define themselves as companies and not as charitable institutions, so that cost-covering work is becoming increasingly important. In addition to economic factors, political and social factors also play a role. For the employees, this is felt above all in the shortage of personnel as an indirect burdening factor. General conditions 1) Institution/the hospital • Many employees, visitors, students on ward • Noise (patient emergency call, monitors …) • Logistic problems (long distances on the ward and to functional areas) • Lack of flexibility of the institution • Questioning participation in training courses 2) Political/social (macrosystem) • Position key • Demographic changes • Loss of image and stigmatisation of care • Inclined position in hospital financing	So with us it’s the doorbell system and the alarms in general. It’s just a huge stress factor; I notice it when I come back from vacation and it doesn’t take me an hour to get that agitated again. Before I had a level of relaxation. That beeping all the time and that red light…it’s just very uncomfortable and put people under pressure and stress. (RN_FG1) All the things that have been mentioned now, you have to try to sort them out, but I think the staffing. If it’s good, is probably due to economic reasons, just as it should be in the OR and intensive care unit, that it makes money, or at least that what makes money compensates, that’s what’s best staffing. (RN_FG1)
	C2.4 Lack of gratification	Lack of reward, appreciation or feedback by supervisors, physicians and patients. As recognition, the thanks for favours and accommodating behaviour, higher salary and joint activities are perceived.	But it’s very stressful that you feel you’re doing a great job and managing it quite well, while the level above you is busy with other things or doesn’t realize it. (SU_I7)
	C2.5 Lack of social support		
	Interprofessionality	Divergences between several professions with effects on the working climate Development toward larger teams and anonymity of the individual employee Low sense of community/commitment Low compliance Poor culture of error Lack of openness and communication problems Hierarchical thinking Authoritarian tone Self-esteem increase at the expense of colleagues in nursing Conflicts (clarified via hierarchy or carried out in the whole team) Professional divergences Quality vs. quantity	B6: Our own doctors do not see the effort. “We’re going for a CT [computed tomography] scan”—with someone who has an extracorporeal procedure, who is ventilated. I can’t go fast. Sometimes it takes me an hour to prepare. And then the doctor comes and says: “Is everything ready?” And pushes the bed in and says, “Bye.” Those are the moments when the appreciation is gone, too. […] B6: Or exactly the same topic with the inexperienced doctors. We have 8 h of high care and high tech. And sometimes 16 h of low care and low tech, because the medical side is simply weak. Yes, and the nursing staff compensates everything because they simply have the know-how. (SU_FG2)
	Group cohesion	Deficits/difficulties in communication, goal orientation, task management, cohesion, assumption of responsibility, etc. within the nursing sector *Task cohesion (task-oriented cohesion) or vertical cohesion (group members for leadership)* • Compensation for management deficits • Allegations of low motivation to perform • Difficult cooperation with superiors • Unclear expectations of superiors regarding MA *Social cohesion (social cohesion) or horizontal cohesion (group members among themselves).* • Conflicts, lack of critical faculties • Bad mood • Unreliability of colleagues • Negative attitude • Lack of commitment among colleagues, loner • High social pressure • Low esprit de corps (triple win) • Rotation of the RNs	And that’s why I sometimes don’t understand, and that sometimes makes me very sad, that this professional group is actually, in my opinion, so miserable. And in fact, so generally miserable. It’s important that you simply have exhausting days in individual situations and that you are allowed to say that, I think. So, there is such a thing. Yes, that’s completely legitimate. I don’t want to be misunderstood. There is every day, but I believe that in every place, in work situations, in care as in other areas, where people say, “Goodness gracious, if I go home today, that was not a good day. It just didn’t go very well. Or I didn’t manage to do everything I wanted to do.” But here in Germany, nursing care is sometimes so thoroughly miserable. And I regret that very much. And that should go better. I: The presentation to the outside. B: Yes. Your own. But on its own. So sometimes I have the feeling that public relations, positively representing the profession, is one thing. But people must also be convinced of it themselves. And I miss that a little bit. Yes, I miss that a bit. (SU_I7)
C3 Resiliencies, strategies and resources	C3.1 Behavioural	Actions to reduce the physical and psychosocial workload; changing the behaviour of an employee, adaptation of the individual/employee to the environment • Supervision, ward and case conferences • Knowledge acquisition and development • Sport/movement • Read • Nature, animals, fresh air • Homeopathy • Healthy eating • Mindfulness • Relaxation trainings (Progressive muscele relaxation…) • participation in occupational health management or reintegration measures • Listen and make music • Religion and spirituality (support through community and rituals) • Massages • Daily structure and breaks • Adjusting job volumes and duty periods • Sick leave/rehab • Change of job within the institution • Change of job outside the institution and resignation	With me it is now the choir, so I think you need something for body and soul. For me it’s just the singing. (SU_I9) And when I was again at the staff council and the actual situation came to light, it was turned off within a day, this open-end working. (RN_I6)
	C3.1.1 Cognitive	• Reframing/reevaluation of critical situations, interpretation • Reframing of the priorities (one by one…) • Relativize basic convictions (high demands) • Segregation from work • Analysing, correcting and organizing/planning work processes and loads • Weighing up the advantages and disadvantages of the profession • Correction of the value system • Delegate responsibility • positive appreciative attitude • Reactance • Focus on yourself and your own health • Positive self-instructions • Higher weighting of the appreciation by patients • Service to rule	Or I start working on myself: Do I still enjoy this profession so much that I can live with it; that I can easily leave out things that the patient might like to have but which he doesn’t need to survive? There are a few luminaries who have done a good job. They’ve always been my role models. Even in intensive care, for example, there was the giant step: always one for two patients, in times of emergency you have to look after three. What a fuss! “I can’t do this at all, I can’t work like this!” I’ve seen nurses who just said very calmly: “What do people need?” He is ventilated, he must get off the respirator. So I wean [practice getting off the ventilator] him. And I mobilize him, and I give him antibiotics. I don’t need to wash him. I need to see if his skin is intact. But whether or not he’ll be walking around outside again in 6 months’ time doesn’t depend on whether I wash him today, but whether he learns to breathe and walk by himself again. That’s reasonable. There were people who were excellent at it. And they were simply my role models. And that it was great fun for me to rethink things. […] And you need a bit of structural thinking so that you don’t get stuck on your little things. (SU_I4)
	C3.1.2 Social	• Social support within the team and outside the clinic (family, friends, religious community) • Clarification of problems and expectations • Exchange with colleagues from other stations • Team activities outside the clinic • Appreciation of the patients	Talk to the wires over there about team effort here. Regularly going out for dinner once in a while, even in your spare time. Or here offers or here, because I think the culture on a station also plays an extremely important role. That you get along well with each other, that you do something together. I think that sometimes it comes too short. But such a structure, then you also go through difficult times together. And then it’s simply easier when you have a bit of a culture in the team. (SU_I8)
	C3.2 Relational	Actions to reduce the physical and psychosocial workload; change of conditions, adaptation of the environment to the individual/MA • Bed reduction • Survey on burdens • Digitisation • Drawing on experience • Auxiliary staff and ‘Triple Win’ • Transferring work areas to external providers • (On-call) standby service • Introduction of additional shifts/wiping services • Involve the Staff Council	So, I, from my point of view, see that there is a relief, even if it is felt differently. We have care assistants, we have team assistants, we have ambulance drivers, we have service staff who distribute the food, we have a lot of support on the outside, we have a growth of the nursing homes. (SU_I9)

Rule: concrete consequences of economisation/personnel shortage are named; facts or description of the circumstances of economisation/personnel

#### C1 Intrapersonal risk factors/predispositions and health complaints (93 codes)

Category 1 summarises the internal cognitive and behavioural factors as well as health complaints and preexisting diseases related to occupational stress.

##### Common factors

As intrapersonal risk factors, both groups described the perfectionist demands of nurses on their work performance, even under situationally poorer working conditions (overcommitment). Furthermore, they suggested limitations such as low problem solving ability, low planning ability, and a low level of reflection and self-confidence and self-efficacy, which leads to self-handicapping, fear of failure, and disorganisation of the work area. Both groups reported that the main focus of nurses’ lives was the profession, stylised into vocation, which results in physical and mental health complaints of the nursing staff. Both groups reported an external locus of control and generalised rejection (resistance to change, defiance) and reactance as further cognitive factors of stress.

##### Distinctive factors

RNs named dysfunctional coping strategies of nurses, such as catastrophizing, generalisation and devaluation of SU as a person and in their function. The RNs blamed the supervisors for the nurses’ low self-confidence, as they felt insufficiently involved, less informed and provided too little room for manoeuvre. The ‘serving nurse’ was described by the RNs as an internalisation of social expectations of nurses. The RNs described themselves as having above-average empathy and high compassion for patients. They described predominantly mental health complaints. SUs described nurses’ high sense of responsibility and lack of ability to distance. SUs focused on physical complaints of the nursing staff (*n* = 16, 64%) while RNs focused on psychological constraints (*n* = 11, 84,6%) (see Table 3 for further details).

**Table 3:** Physical and psychological constraints

	RN		SU		TOTAL	
	N	%	N	%	N	%
Physical						
Sleep disorders	2	33.3	4	66.6	6	24.0
Joint pain (shoulder, hip, knee)	1	25.0	3	75.0	4	16.0
Back pain	4	80.0	1	20.0	5	20.0
Irregular and unhealthy diet	2	100	0	0	2	8.0
Herpes zoster	1	100	0	0	1	4.0
Varices	1	100	0	0	1	4.0
Obesity	2	100	0	0	2	8.0
Migraine	1	100	0	0	1	4.0
Preexisting conditions (arterial hypertension, multiple sclerosis, rheumatism)	2	66.6	1	33.3	3	12.0
Total	16	64.0	9	36.0	25	100
Psychological						
Inner restlessness	1	100	0	0	1	7.69
Resignation/reactance	1	20.0	4	80.0	5	38.46
Tiredness/exhaustion	0	0	4	100	4	30.77
Problems of concentration	0	0	1	100	1	7.69
Depressive mood	0	0	1	100	1	7.69
Compassion fatigue	0	0	1	100	1	7.69
Total	2	15,4	11	84,6	13	100

#### C2 External risk factors/environmental conditions (284 codes)

Category 2 summarises external factors of high job demand, lack of autonomy, economical and relational factors, lack of gratification and social support.

#### C2.1 High job demand (119 codes)

##### Common factors

With regard to high job demands, both groups described external factors of high workload due to a significant reduction in the length of stay of patients, high patient turnover rate, and increased time pressure with an increased demand on the quality of care (especially medical care) at the same time. Both groups also described challenging patient contacts (language barriers, fates, losses, excessive demands on care), new organisational structures (external service providers taking over areas of patient care), staff shortages, and spatial conditions such as long walking distances and being on call as further burdens,. Both groups suggested that nurses did not differentiate between private (empty-nest syndrome, caring for relatives, part-time jobs in nursing and voluntary work) and work-related stress and that nurses tend to attribute the accumulation of stress to work.

##### Distinctive factors

On one hand, RNs referred to a lack of knowledge and, on the other hand, further training to improve knowledge outside working hours as additional stressors. Among workload factors, RNs included high documentation effort, shift work and overtime without breaks, induction and supervision of new nursing staff, short-term changes (e.g.,., helping out on wards with staff shortages) and verbal and physical attacks by colleagues or patients. SUs described the handover of tasks from physicians, nursing colleagues or supervisors unrelated to the core responsibility of nurses (e.g., the induction and supervision of medical colleagues) and adaption to high- and low-care quality as burdens.

#### C2.2 Lack of autonomy (24 codes)

##### Common factors

Both groups saw the heteronomy of physicians, supervisors, functional departments, and other wards (e.g., in-patient transfers) as a stressor, because they require interruptions and adjustments in the nurses’ work processes. A lack of information, transparency and openness were mentioned as conditions for experienced helplessness and loss of control.

##### Distinctive factors

RNs reported that nurses feel pressure to justify themselves to SUs for mistakes and failures to complete the workload. RNs mentioned experiencing arbitrary rostering and withholding of further training by SUs as sanctions. They described a low level of participation and disinterest of SUs in their opinions. Some SUs reported that supervisors suggest job insecurity to young professionals to force dependency.

#### C2.3 Economical and relational factors (63 codes)

##### Common factors

Staff shortages, outage management and changes in regular staffing were considered to be a consequence of the conditions of the political framework, specifically the slanting position of hospital financing as a condition for workload. Because of the focus of profitability in hospitals, quantity came at the expense of quality of care. The public opinion on work performance and even stigmatisation of nursing due to mainly reported insufficient payment and poor quality of care was seen as the main reason for the shortage of young professionals.

##### Distinctive factors

Some RNs saw the lack of staff as a direct threat to the quality of patients care and the stress of their colleagues. Furthermore, crowds (e.g., visitors, hospital staff, external service providers) on the wards, noise (e.g., patient bells, telephones), lack of equipment and logistical difficulties (e.g., lack of space) are factors that make work difficult. Mergers and consolidation of clinics have a negative effect on stress level. RNs believed that the employer is interested only in the workforce and not in the employees’ health. The current tendency of supervisors to present the nursing profession positively in the media was perceived as a trivialisation of the work situation and interpreted by RNs as a glorification and devaluation of nursing work.

#### C2.4 Lack of gratification (26 codes)

##### Common factors

Nurses’ high moral and performance standards were linked to a high need for appreciation, which was reported as rarely experienced by nursing staff.

##### Distinctive factors

RNs were eager to receive appreciation of performance as thanks for concessions and primarily desired salary increases, approval of further education and training (considered as working time), personal contact sought through their superiors, and showing interest in nurses’ work. If there is a lack of appreciation from their supervisors, then, according to RNs, appreciation is sought through colleagues and patients. A lack of gratification and resignation was suggested as a cause for absenteeism. According to RNs, experienced employees should receive above average recognition from supervisors. Some SUs admitted that supervisors were too exhausted to give positive personal recognition to each individual employee, and appreciation by physicians was suggested to be more important for nurses than appreciation by supervisors.

#### C2.5 Lack of social support (100 codes)

##### Common factors

Interprofessional distrust, lack of openness and low esprit de corps were described in terms of interdisciplinary cooperation and a lack of group cohesion within nursing teams. Both groups described nursing staff as frustrated and complaining (“general lament”) and reported that team spirit was depressed. Nurses concentrating only on the negative aspects of teamwork can infect colleagues with a hostile attitude, which can lead to poor cohesion. Openness, trust, conflict management and dealing with mistakes were described as needing development. Language barriers among employees with a background of migration prevent a good integration of new employees. In the course of the high workload, the nurses hardly have time for interpersonal interactions.

##### Distinctive factors

RNs reported interprofessional communication problems with predominantly professional differences of opinion. Criticism was not directed at the nursing staff but rather through their superiors. Frequent changes of physicians prevent their integration into the team. Some SUs reported seeing in recent years a development toward larger and more heterogeneous teams, without agreements and compromises, especially with the medical service. SUs described a hierarchical relationship between nurses and physicians. Conflicts usually remained unspoken, without interdisciplinary clarification. Cooperation with other departments or units was reported as being sometimes inflexible and rigid; thus, nurses tended to interpret the behaviour of other professions and departments as stimulation of self-esteem at the expense of the nursing team.

##### Horizontal^2^ group cohesion

RNs evaluated the tendency of prioritizing private life as “laziness” and “unreliability” and as closely connected to absenteeism, for which colleagues must compensate. Lack of interpersonal exchange leads to a deterioration of relationships and commitment and even to anonymity in the nursing team. Some SUs saw a poor working atmosphere as a result of differences in nurses’ motivational levels.

##### Vertical^3^ group cohesion

RNs reported fluctuations of supervisors and leadership deficits as factors that made work difficult. Solutions proposed to supervisors by nursing staff were not implemented as expected, so nurses felt that they were not taken seriously and were devalued. According to RNs, the communication of facts via supervisors seemed selective through distortion or misreporting to the disadvantage of nurses. If nurses express dissatisfaction or criticism, negative consequences, such as termination of the employment contract, have sometimes been implied. Some SUs described experiences in which supervisors rose above nurses and discredited them in their absence. SUs also reported unclear expectations of supervisors in nurses.

#### C3 Resiliencies, strategies, and (job) resources (217)

Category 3 summarises the behavioural, cognitive, social, and relational measures or actions used to protect from or to reduce physical or psychosocial workload or distress.

#### C3.1 Behavioural strategies (54 codes)

##### Common factors

Both groups reported individual strategies for actively switching off and relaxing, such as relaxation techniques, sports, outdoor activities, and contact with animals in leisure time outside the clinic. Further education and multiplication of knowledge in the team were seen as stress-reducing measures. To reduce stress, both groups described strategies such as self-induced job changes, job rotation or termination of employment in addition to a reduced job volume.

##### Distinctive factors

RNs reported individual resources, for example, sleep-improving measures and maintaining the daily structure despite shift work as coping strategies as well as participation in occupational health management measures such as psychological consultations and the use of the peer support of the in-house crisis service. However, RNs assumed these strategies lead only to a short-term reduction in dissatisfaction and stress. Some SUs reported that nurses communicated their grievances to the public as a coping strategy. On the contrary, SUs suggested that the negative medial promotion of the nursing profession enlarges the problem of filling vacant positions; staff shortages and the individual workload remained unchanged, and the long-term relief of the employees was not forthcoming.

#### C3.2 Cognitive strategies (63 codes)

##### Common factors

Both groups stated that to improve job satisfaction, it is essential to develop an appropriate inner attitude toward self-care, ego relation and solution orientation, with a medium sense of responsibility and openness for new things. Both groups considered it necessary to rethink and reorganise individual work processes and their own priorities (e.g., delegating). In addition, both groups believed that reframing the employees’ experiences and demands seemed to provide a chance to highlight positive aspects, give meaning and significance and adapt demands realistically to the circumstances. Both groups reported strategies of emotional distancing, such as the decision to refuse requests and tasks, as stress-reducing measures.

##### Distinctive factors

Some SUs described optimism, basic trust, a sense of community and serenity (e.g., in enduring aversive states) as stress reducing. In the opinion of SUs, reflecting stress factors by revising basic assumptions about responsibility would promote health. With cooperation between nurses and supervisors, SUs would like to experience a separation of person and function from nurses.

#### C3.3 Social strategies (34 codes)

##### Common factors

Both groups described joint discussions with management about team measures, dissatisfaction and the balancing of mutual expectations as helpful. In addition, social support (e.g., joint leisure activities with colleagues after work) as well as a social network outside the clinic (friends and relatives) were mentioned as being conducive to well-being.

##### Distinctive factors

Some SUs considered the exchange with colleagues from other departments as helpful. They advocated democratic decision making within the team. Some reported that especially not talking about work with family members was relieving.

#### C3.4 Relational strategies (66 codes) 

##### Common factors

Both groups suggested a regulated break structure as part of the ward routine as directly regulating stress. Both groups reported on measures such as supervision and coaching, as well as on the possibility of extending professional competence by internal and external further training measures and flexible working hours.

##### Distinctive factors

On one hand, relational changes were seen as a burden (e.g., digitalisation, reduction of treatment places, etc.); on the other hand, they were also seen as an opportunity for relief (e.g., two-shift system, the introduction of short and intermediate shifts, time-out/cures/rehabilitation with the support of the employer, etc.). SUs reported factors to ease the burden, such as a variety of skills exhibited by team members (e.g., wound management, port catheter exchange), nursing assistants, and nursing migration, which allows nurses to delegate and hand over work areas. Ward meetings and working groups as well as one-on-one meetings with supervisors and the support of the staff council were further elements that could improve individual job satisfaction. Special management coaching sessions had been set up for supervisors. SUs suspected that an expansion of the employees’ scope of action could help to relieve the workload.

## Discussion

The aim of this study was to investigate and compare stress and resilience factors in the nursing profession from the perspective of registered nurses and their supervisors. To systematize the results, we used the job demand and resources model [15] and integrated it into the Lazarus model [32] in order to emphasize the relevance of cognitive evaluations in the assessment of demands and resources in the development of stress. We found a large agreement in terms of stress and resilience factors, but there were also disagreements and contradictions in causal relationships and accountability for resolving situations. To discuss the results in a clear and comprehensible way, job demands and stress factors are discussed first, followed by the job stress-reducing and resilience factors with regard to similarities and differences between nurses and supervisors. Finally, we discuss the issue of responsibility and the shortfall in the needs of nurses.

### Job demands and stress factors

We divided stress factors into internal and external factors (including high job demand, lack of autonomy, gratification, social support), economical and relational factors. The section of internal–cognitive factors showed great agreement between registered nurses and supervisors, especially in overcommitment, external locus of control, low self-efficacy and unplanned behaviour. Both nurses and supervisors evaluated the overcommitment of nurses, that is, hard work and long hours to meet or even exceed the work requirements, as a risk factor for work-related stress. Previous studies in the literature have shown that overcommitment and overidentification have an (indirect) effect on stress, burnout [39], anxiety [40], and depression in nurses [41]. However, in contrast to supervisors, nurses highlighted overcommitment as an internalisation of society’s image of the “serving nurse” and a consequence of high work demands, applied by supervisors (resp. by institution).

In addition to overcommitment, participants considered external locus of control, low self-efficacy, and unplanned behaviour as stress factors. These findings are in line with empirical studies confirming the correlation between locus of control, self-efficacy and burnout [42].

With regard to external stressors, next to high job demands and staff shortage, both groups consistently reported a low esprit de corps within the team and supervisors and low interprofessional cooperation. They suggested that nursing staff often experienced either uncertainty or multiplicity of demands, goals or expectations directed at them because of the flattening of hospital hierarchies. These descriptions can be understood as role ambiguity [43], which is closely related to role conflicts [44]. Role conflicts in turn contribute to high emotional exhaustion [45], reduced well-being [46], job dissatisfaction, low commitment, and high intention to leave the profession [47].

Some nurses pointed out that internal and external stressors influence each other. They reported internal factors as mediators of external stress factors, such as low autonomy, participation and gratification. This is in line with the findings of previous studies, in which professional autonomy was identified as an important predictor of job satisfaction [48], work performance and organisational commitment in nurses [49]. An imbalance between efforts and rewards as a reflection of insufficient gratification was positively related to emotional exhaustion [50].

Overall, nurses and supervisors largely agreed in their assessment of internal and external stress factors. Thus, it can be assumed that the stress of nurses is adequately communicated to supervisors and that supervisors are aware of the stress situation of their followers. This understanding for the employees could have a positive influence on SSR and the social work environment and may buffer nurses’ psychological distress and increase their well-being [21].

### Job resources and resilience factors

RNs and SUs agreed to a large extent about nurses’ behavioural, cognitive, social and relational resiliencies and coping strategies, with highest congruency in behavioural aspects and lowest in social support. With regard to behavioural resiliencies, RNs and SUs in our study reported going into action as helpful (e.g., presentation of nurses’ job situation in the media such as Twitter and Facebook to force awareness of the publics’ and stakeholders’ attention to their situation). Although the activity of the nurses was appreciated by the supervisors, some of them were critical of it, saying that this activity has a negative influence on the population’s attitude toward the nursing profession. They suggested the profession might become less attractive for later applicants, as also described earlier by Donelan et al. [51]. The media portrayal of nurses as “victims of serious workplace problems” [52] seems to be an external risk factor, as it can increase the staff shortage and worsen the relationship with inpatients [53].

With regard to cognitive coping strategies, both groups evaluated cognitive skills such as adaptation ability, solution orientation and reflective ability as highly protective. Looking at the differences, supervisors identified further cognitive skills in dealing with work-related stress. They mentioned the adjustment of performance expectation to the circumstances and the knowledge of sufficient coping strategies. Previous research related both coping strategies directly to nurses’ resilience, in accordance with Cam [54].

Both groups agreed that social support and positive professional and private contact with colleagues and supervisors can be a resource in dealing with work-related stress, as confirmed in previous studies [21]. Good relationships in the workplace, especially with supervisors, have an impact on the well-being of nurses [4]. Keeping its high impact in mind, social support can be promoted through good contact management (e.g., a high level of participation, joint activities with the department).

With regard to relational measures and strategies, both groups agreed that recreational phases during working hours were helpful. In addition, both groups saw support from the employer in the form of supervision, coaching and further training as stress-reducing measures; however, they reported that, to be able to better reconcile family and work, they would also like to see more flexibility in working time. Recent studies support this claim: individual-oriented working time flexibility was positively related to employees’ work–life balance, whereas organisation-oriented working time flexibility had a negative effect on them [55]. There was disagreement with regard to the structural changes that, according to the supervisors, would lead to relief in the long term but would be perceived as a burden by the nursing staff in the short term. This phenomenon may be related to a lack of participation in the decision-making process and thus reduced commitment of the nurses. Shared decision making can improve shared governance as collaborative decision making between nurses at every level and can strengthen the nurse workforce, improve job satisfaction and ultimately improve the quality of care [56]. Therefore, establishing a participative leadership style and early involvement in decision making could increase nurses’ sense of control, and short-term aversive consequences could be contextualised with positive long-term consequences, making it easier for staff to accept them. However, this remains speculative and should be the subject of further investigations.

### Measures to improve the situation are experienced as insufficient

Implemented measures such as the establishment of projects for the migration of nurses, the deployment of medical assistants, changes in conditions (e.g., merging wards, occupational health management) and expansion of nurses’ scope of action were well intended but seemed to have bypassed the registered nurses’ needs and were rather seen as further stressors by nurses because of the necessary adjustment efforts.

The supervisors in our study assumed that measures for the expansion of nurses’ scope of action and area of responsibility, such as training new employees and deciding on the priorities of tasks to be completed, could reduce power struggles, uncertainties and stress. However, members of the nursing staff experienced the shift of responsibility from the middle management level to followers as reinforcing or increasing uncertainties of responsibility and decision-making authority. A previous study reported that an expansion of responsibility with limited autonomy and lack of career opportunities caused dissatisfaction and conflicts, as further stress factors of nurses [49]. Consequently, to increase job satisfaction and reduce work-related stress, an expansion of responsibility should go hand in hand with an expansion of the scope for action.

As a further example of bypassing the needs of nurses, nurses reported stress due to a lack of knowledge, an assumption not shared by supervisors. Training opportunities to improve knowledge and reduce stress are offered mostly after working hours, which, as nurses reported, would in turn lead to further stress. Empirical studies have shown a divergence between the level of knowledge, attitudes and nurse practice [57] and revealed a negative correlation with stress [58]. Therefore, educational programmes presumably would have a negative effect on the experience of stress and a positive influence on work performance. Offering those programmes during working hours may decrease stress and would not generate additional workload.

### Responsibility for nurses’ health

Overall, nursing staff and supervisors predominantly agreed regarding their assessments of risk and protective factors of stress, and supervisors showed an understanding of the situation of their followers. In our interviews, nurses made support needs (individual contact, work facilitation, further training and gratifications) clearly visible but tended to hand over the responsibility for psychological well-being and needs to their supervisors and therefore put supervisors and the institution in charge. Conversely, supervisors also returned the responsibility of mental health to nurses. For example, in our study, nursing staff associated the required subordination of their work processes with a lack of supervisor appreciation, which would cause mortifications that resulted in low self-confidence and compensatory overcommitment (see the internal factors above). Nursing staff and supervisors differed with regard to the provider of appreciation. According to supervisors, appreciation should come from the person who caused the injury, not automatically from supervisors or nursing staff. Although the negative influence of low appreciation on job satisfaction [59] and burnout [60] is well known, the question of who must provide the appreciation has not yet been sufficiently investigated.

These results provide a reason to reconsider procedures in practice, research and training approaches. First, it should be positively emphasised that there is little difference in the perceptions of nurses and supervisors, suggesting that there is an awareness of the situation of the nurses. The differences mentioned might be based on the interindividual differences of the nurses that were unknown or not salient to the supervisors. It can be helpful for both parties to take the perspective of the other party and to be understanding and considerate of the motivations. The fact that we still have to assume that we are dealing with two different parties with different motivations shows that nurses and supervisors are only partially understood as a team. It is this front or camp mentality that makes it difficult to work together effectively for the benefit of the staff. Here direct communication seems to be helpful for bringing this into the awareness of the decision makers and to be able to take it into account in the implementation of relational and occupational (health) measures. However, at the same time, it should not go unmentioned that employers and supervisors cannot take into account all of the negative consequences and individual circumstances of the nurses. Rather, this requires a compromise between both parties—a certain flexibility on the part of the nurses in taking up and implementing the relational and occupational (health) measures but also on the part of the institution in implementing the measures. Because of incongruent perceptions, expectations, and disagreements, it is necessary that both groups remain in close contact and exchange with each other to minimise stress for the nursing staff and to improve cooperation.

As implications for further research, quantitative and empirical studies should be applied to verify stress and resilience factors from the perspective of nurses und supervisors. Because we conducted the study at a maximum care hospital, a comparison with hospitals of other care levels is necessary to verify the validity of the data.

In addition to implications for practice and further research, there are also implications for the development of training and education programmes by including the above-mentioned factors in the contents. The content of the training should be adapted to the modern way of working and supplemented with the promotion of personal responsibility (self-development). This means that, in the future, to promote nurses’ self-efficacy and self-confidence and to enable them to use participative offers, modern training should focus on strengthening the resources and skills of nurses on a cognitive, behavioural and emotional level. We hope that the development of excellent training courses for improving the health of nursing staff will continue, to once again make this indispensable and enriching profession more attractive and health conserving.

### Limitations

This study has several limitations that should be considered. First of all, the self-selection bias should be mentioned. Although we attempted to draw a broad representative sample, it is possible that participants triggered by the topic in particular registered. The second point is a possible social desirability bias, as participants were familiar with the topics of stress and stress management. This escalated into a controversial topic in the media at the time and the pressure to conform was so high that dissenting opinions were often not voiced, especially in focus groups. The short duration of the focus groups may have favoured this. The third point concerns generalizability. The participants were employees of a university hospital, so the statements are valid for this setting. Generalization to other work settings must be examined. Fourth, as our data is from 2018, other important risk and stress factors related to the Covid-19 pandemic were not taken into account. As this is the first study on the risk and protective factors of stress from different nursing hierarchy levels and reveals the first important results, we hope to inspire future quantitative work on cross-hierarchy stress-related phenomena to verify the observed phenomena.

## Conclusion

To the best of our knowledge, this is the first study to compare the factors influencing nurses’ job stress and resilience from the perspective of both nurses and supervisors. There was broad agreement between the two groups. However, there was also disagreement between nursing staff and supervisors, indicating that they should enforce close exchanges to improve mutual understanding. Furthermore, measures to meet nurses’ needs to minimise stress and improve collaboration and job satisfaction should be developed closely together (e.g., practising participative leadership). A focus should be placed on restructuring training and education programmes with supplementation of promoting personal responsibility (self-development).

## Supplementary Information

Supplementary Material 1.

Supplementary Material 2.

## Data Availability

The datasets used and analysed during the current study are available from the corresponding author on reasonable request.

## Abbreviations

RN: Registered nurses
SU: Supervisors
SSR: Supervisor–subordinate relationship

## Footnotes

1 German registered nurses have no managerial position. Ward nurses and nurse managers are registered nurses with management responsibility, ward nurses for one ward and nurse managers for departments or a group of wards. Nurse managers are superior to ward nurses, which are superior to registered nurses.

2 Group cohesion at one hierarchical level.

3 Group cohesion across several hierarchical levels.
