# Supplementary material for: Nursing staff and supervisors perceptions on stress and resilience: a qualitative study
Source: BMC Nurs. 2025 Jan 22;24:76. doi: 10.1186/s12912-025-02712-x (PMC11756110; doi:10.1186/s12912-025-02712-x)
Supplement: Supplementary file 1 — Supplementary Material 1. [file 12912_2025_2712_MOESM1_ESM.docx]

# Appendix A: Interview Guide

## Work situation

- Please describe your workplace to me!
- How do you/nurses currently feel at work?
  - How do you/nurses evaluate it?
  - What is going well?
  - What is not going well?
- What do you/nurses experience as particularly stressful in your/their work?
- How do you/nurses experience the work over time?
- Which in-house programmes do you/nurses take advantage of (health promotion, other offers . . .)? What do you wish for; what is missing?
- With regard to your/nurses health:
  - What restrictions do you experience with nursing staff?
    - Changes?
  - How do you/they deal with them?
  - Which strategies are helpful?
- What motivates you/nurses in your/their work?
- What demotivates you/nurses in your/their work?
- How would you/nurses describe the culture within your ward?
- In terms of:
  - Openness
  - Recognition/appreciation/direct feedback
  - Trusts
  - Dealing with conflicts/error culture
  - Teamwork

## Professional biography:

- What has changed during your/nurses’ professional career? (the most important aspects, individual, organisational, work, knowledge . . .)
- Supervisors only:
- How do you experience working with nurses?
- What is important to you in cooperation?
- What is important to you when hiring an applicant, or what are the relevant criteria?
